# Insulin-like growth factor 2 axis supports the serum-independent growth of malignant rhabdoid tumor and is activated by microenvironment stress

**DOI:** 10.18632/oncotarget.17617

**Published:** 2017-05-04

**Authors:** Ting Li, Jin Wang, Pengfei Liu, Jiadong Chi, Han Yan, Lei Lei, Zexing Li, Bing Yang, Xi Wang

**Affiliations:** ^1^ Department of Cell Biology, 2011 Collaborative Innovation Center of Tianjin for Medical Epigenetics, Laboratory of Epigenetics in Development and Tumorigenesis, Tianjin Research Center of Basic Medical Sciences, Tianjin Key Laboratory of Medical Epigenetics, Tianjin Medical University, Tianjin 300070, China; ^2^ Department of Lymphoma, Sino-Us Center of Lymphoma and Leukemia, Tianjin Medical University Cancer Institute and Hospital, National Clinical Research Center for Cancer, Key Laboratory of Cancer Prevention and Therapy, Tianjin's Clinical Research Center for Cancer, Tianjin 300060, China; ^3^ Department of Head and Neck Oncology, Tianjin Medical University Cancer Institute and Hospital, Tianjin 300060, China

**Keywords:** malignant rhabdoid tumor (MRT), insulin-like growth factor 2 (IGF2), insulin-like growth factor 1 receptor (IGF1R), IGF axis, microenvironment stress

## Abstract

Malignant rhabdoid tumors (MRTs) are rare, lethal, pediatric tumors predominantly found in the kidney, brain and soft tissues. MRTs are driven by loss of tumor suppressor SNF5/INI1/SMARCB1/BAF47. The prognosis of MRT is poor using currently available treatments, so new treatment targets need to be identified to expand treatment options for patients experiencing chemotherapy resistance. The growth hormone insulin-like growth factor 2 (IGF2) signaling pathway is a promising target to overcome drug resistance in many cancers. Here, we evaluated the role of IGF2 axis in MRT cell proliferation. We showed that microenvironment stress, including starvation treatment and chemotherapy exposure, lead to elevated expression of IGF2 in the SNF5-deficient MRT cell line. The autocrine IGF2, in turn, activated insulin-like growth factor 1 receptor (IGF1R), insulin receptor (INSR), followed by PI3K/AKT pathway and RAS/ERK pathway to promote cancer cell proliferation and survival. We further demonstrated that impairment of IGF2 signaling by IGF2 neutralizing antibody, IGF1R inhibitor NVP-AEW541 or AKT inhibitor MK-2206 2HCl treatment prevented MRT cell growth *in vitro*. Taken together, our characterization of this axis defines a novel mechanism for MRT cell growth in the microenvironment of stress. Our results also demonstrated the necessity to test the treatment effect targeting this axis in future research.

## INTRODUCTION

Malignant rhabdoid tumors (MRTs) are rare, highly aggressive neoplasms that primarily develop in infancy and early childhood arising from kidney or extra-renal sites such as brain or soft tissues [1]. MRTs are characterized by biallelic alteration of the *SMARCB1/INI1/SNF5/BAF47* tumor-suppressor gene, which encodes a core component of the chromatin-remodeling complex SWI/SNF [2, 3]. Despite the existing standard intensive multimodal therapy, the long-term survival rate of MRT patients is less than 30% [4, 5]. The poor prognosis is due to high cellular proliferation, propensity for metastasis and resistance to radio- and chemo-therapy [6]. However, the mechanisms of MRT survival in poor environment remain largely unknown.

The insulin-like growth factor 2 (IGF2) is a 7.5 KDa mitogenic peptide hormone produced mainly by the liver, but also secreted by tissues where it acts in an autocrine or paracrine manner [7]. IGF2 is a major growth factor in fetal development, its mRNA expression is down-regulated postnatally in kidney and liver [8]. The IGF axis is a complex signaling network, composed of peptide-ligands IGF1, IGF2 and insulin, and receptors IGF1R (insulin-like growth factor 1 receptor), IGF2R (insulin-like growth factor 2 receptor), INSR (insulin receptor), as well as IGFBPs (IGF binding proteins) [9, 10]. IGF2 has similar affinities for the IGF1R and the short isoform of the INSR (IR-A). This hormone signals through both IGF1R and INSR, activating downstream signaling to promote cell growth [11]. Unlike IGF1R and INSR, IGF2R negatively regulates ligand bioavailability and mammalian growth [12]. In addition, IGF2 binds to several IGFBPs that regulate the bioavailability of IGF2 [13]. Evidence shows IGF2 is commonly overexpressed in cancer. Based on data derived from epidemiological studies and experimental models, IGF2 has recently been implicated in drug resistance [14–17]. Treatments that target IGF2, such as ligand-specific antibodies, are showing promise in preclinical studies [18–20].

IGF1R is crucial for tumor transformation and survival of malignant cells. In many tumors, binding of IGF2 to IGF1R inhibits apoptosis and promotes cell proliferation [21]. As an anti-cancer target, IGF1R has become an attractive target for novel cancer therapeutics [22]. Other groups have reported significant IGF1R expression in AT/RT (Atypical teratoid rhabdoid tumor), which are related to MRT and occur in the central nervous system. In their research, treatment of AT/RT cell lines BT12 and BT16 with IGF1R antisense oligonucleotides resulted in a significant decrease in cellular proliferation [23]. The most advanced strategies used have been monoclonal antibodies against IGF1R, and small molecule inhibitors. Some have entered phase III clinical trials for treating human cancer [24]. IGF2 can bypass IGF1R signaling and avoid inhibition by stimulating IR-A, inducing mitogenic signals [22]. In this case, dual IGF1R/INSR inhibition may improve the treatment outcome. In response to the stimulatory ligand IGF2, IGF1R activates downstream RAS/ERK kinase pathway and the phosphinositide-3 kinase (PI3K)/AKT pathway, which are related to cell proliferation and anti-apoptosis [25]. The PI3K/AKT pathway is a central axis in survival and proliferation of SNF5-deficient cells. Eden et al. found aberrant and persistent activation of AKT under low serum conditions was corrected when SNF5 was restored [26]. In many tumors, activated oncogenic signaling, such as RAS, AKT and Myc, contributes to ongoing neovascularization by upregulation of proangiogenic factors [27].

To date, the role of IGF2 in MRT is largely unknown. Here, we sought to characterize IGF2 axis in MRT cells. Poor microenvironmental conditions are a characteristic feature of solid tumors [28]. Work in our laboratory using serum deprivation and chemotherapeutic agents to stimulate MRT cells induced IGF2 overexpression, indicating IGF2 plays important roles in MRT cell proliferation and survival under the microenvironment stress. We found the serum-free growth capacity of MRT cell lines G401 and BT16 is dependent on autocrine IGF2 by using the IGF2 neutralizing antibody. In addition, NVP-AEW541, a small molecule inhibitor of IGF1R, blocked recombinant human IGF2 (rhIGF2) induced AKT phosphorylation, and caused cell death in both G401 and BT16 cell lines. Furthermore, the allosteric AKT inhibitor MK2206 2HCl impaired the growth of MRT cell lines in a dose-dependent manner. Taken together, our data provide evidence that the IGF2 axis plays important roles in cell proliferation and confronting rough environmental in MRT. Therefore, it is worthy to test the possibility of this pathway to be a potential therapeutic target in the treatment of MRT in the future research.

## RESULTS

### SNF5-deficient MRT cell lines G401 and BT16 exhibited serum-independent persistent cell growth accompanied by IGF2 axis upregulation

**Figure 1:**
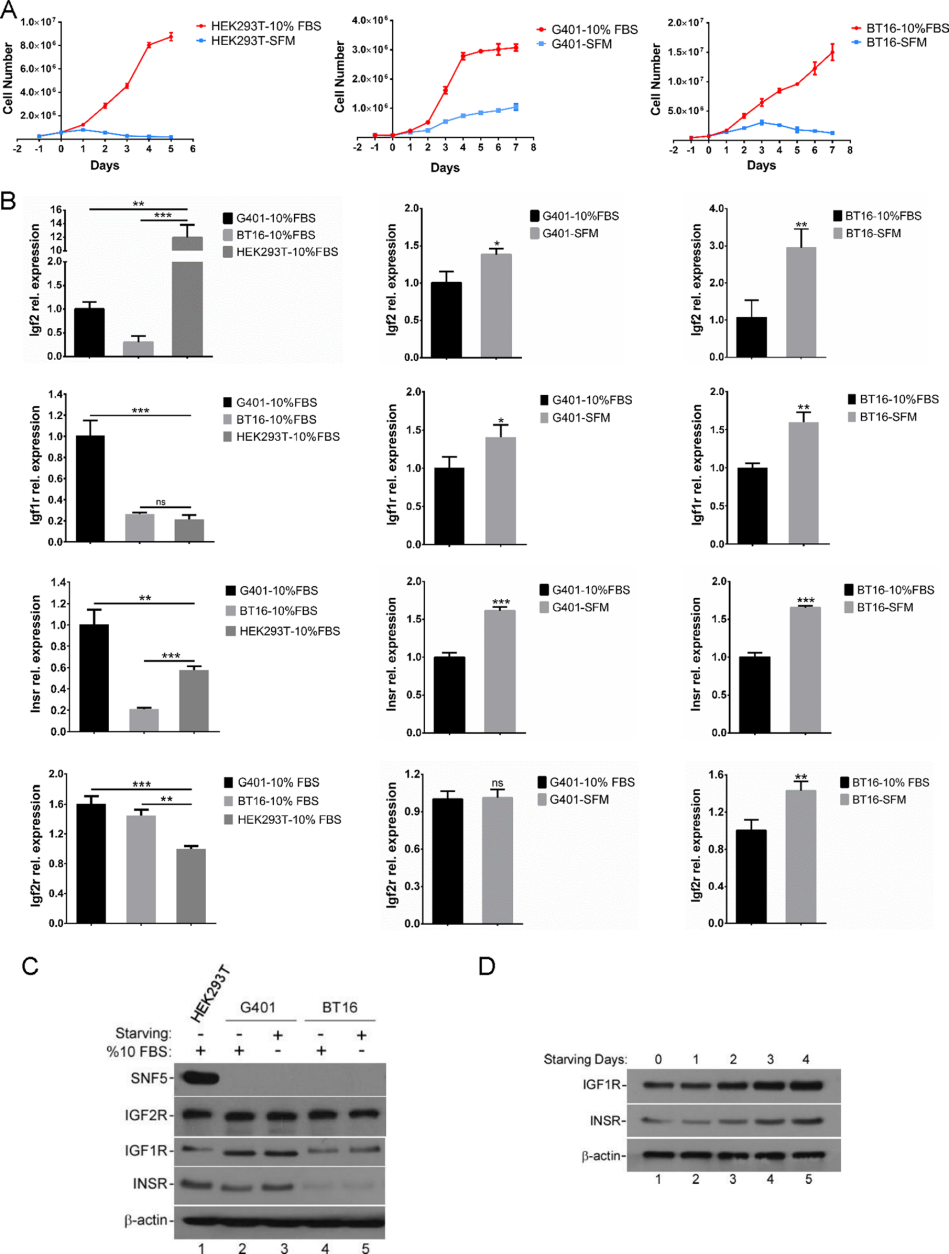
The serum-free growth of MRT cell lines is accompanied by the activation of IGF2 axis (**A**) Growth curve of HEK293T, G401 and BT16 in α/Ham medium containing 10% FBS (-10% FBS) or serum-free α/Ham medium (-SFM). Cells were plated out in 60 mm dishes as described in Methods. After attached overnight, all plates were washed and medium replaced with serum-free α/Ham medium. 24 hours later medium was changed again in the presence or absence of serum. Medium was changed daily for the indicated days. On each day triplicate dishes were counted in a haemocytometer. Data represent the mean ± SD. (**B**) Serum deprivation induced IGF2 axis activation. G401, BT16, HEK293T were cultured with α/Ham medium containing 10% FBS or with serum-free α/Ham medium for 72 hours. The IGF2, IGF1R, INSR, IGF2R mRNA levels, determined by reverse transcription quantitative real-time PCR in three independent experiments, are shown as fold change on the graph. Data represent the mean ± SD. *p < 0.05, **p < 0.01, ***p < 0.001. (**C**) The protein levels of SNF5, IGF1R, INSR and IGF2R in control and starved G401, BT16, HEK293T cells. Cells were seeded into 60 mm dishes. After attachment, cells were cultured for 72 hours with α/Ham medium containing 10% FBS or with serum-free α/Ham medium. The expression levels of SNF5, IGF1R, INSR and IGF2R were investigated by western blot analysis. β-actin served as a loading control. A representative example from 3 independent experiments is shown. (**D**) The expression levels of IGF1R and INSR following serum-free treatment for the indicated days. G401 were cultured with α/Ham medium containing 10% FBS (lane 1) or with serum-free α/Ham medium (lane 2-5) for 1-4 days. Upregulation of IGF1R and INSR were analyzed by immunoblotting. β-actin served as a loading control. A representative example from 3 independent experiments is shown.

MRT is one of the most aggressive pediatric malignancies [29]. Tumor-secreted growth factors affect tumor microenvironment, as well as stimulate the cancer cells to proliferate and develop a more malignant phenotype in an autocrine manner [30]. Therefore, in order to examine the potential for autocrine growth of the MRT cell lines under investigation, we assessed the MRT cell proliferation ability in serum-independent conditions [31]. We found that more than 90% of HEK293T cells died within 4 days in serum-free medium. In contrast, SNF5-deficient cell line G401 exhibited sustainable growth in the serum-free medium for more than 7 days. However, the growth rate was much lower compared with cells cultured in normal conditions. The BT16 cell line showed weaker proliferation in serum deprivation compared with G401. The total BT16 cell number reached maximum at day 3, with some cells surviving more than 7 days after FBS withdrawal (Figure 1A). These data suggest an unknown factor exists to mediate cell proliferation in serum-free medium, best exemplified by G401.

According to the previous report, MRT cells showed highly strong and specific IGF2 mRNA expression, and autocrine IGF2 may contribute to the serum independence of G401 cells [31, 32]. Therefore, we determined whether IGF2 axis showed aberrant activities in starved MRT cell lines. Considering both G401 and BT16 exhibited the highest growth rate on the third day of starvation treatment, we harvested HEK293T, G401, BT16 cells at this time point. They were cultured in α/Ham medium containing 10% FBS or serum-free α/Ham medium, and then analyzed for IGF2, IGF1R, INSR, and IGF2R mRNA expression levels by RT-qPCR. We found the IGF2 expression levels in G401 and BT16 were both upregulated during starvation, although the IGF2 basal mRNA levels in G401 and BT16 were lower compared with HEK293T (Figure 1B). Since IGF2 exhibits homology with insulin, and IGF2 ligand binding may active both the insulin-like growth factor 1 receptor (IGF1R) and insulin receptor (INSR) tyrosine kinases [33], we detected the change in mRNA expression levels of IGF1R and INSR. We found the basal levels of IGF1R and INSR in G401 were higher than BT16 and HEK293T (Figure 1B). Similar to IGF2, the IGF1R and INSR mRNA levels were also upregulated upon starvation, implying increased sensitivity to ligand signaling. The increased expression levels of IGF1R and INSR were also detected by western blot. Consistent with RT-qPCR results, we found the IGF1R and INSR protein levels slightly upregulated in starved G401 and BT16 (Figure 1C). The expression levels of IGF1R and INSR increased in a time-dependent manner from Day 0 to Day 4 (Figure 1D). Unlike IGF1R and INSR, IGF2R inhibits the IGF2 signaling pathway. IGF2R binds IGF2 and functions for trafficking excess ligand to lysosomes for degradation and/or directly mediating IGF2 signaling [34]. We showed here, the mRNA level of IGF2R was increased in serum starved G401 and BT16 cell lines, but the protein level had little changed (Figure 1B and 1C). The absence of SNF5 in MRT cell lines was also confirmed by western blot analysis (Figure 1C). Taken together, these results proved the IGF2 axis was upregulated in MRT cell serum-starvation growth.

### Epirubicin HCl and etoposide induced IGF2 overexpression, and activated downstream survival-related pathways

**Figure 2:**
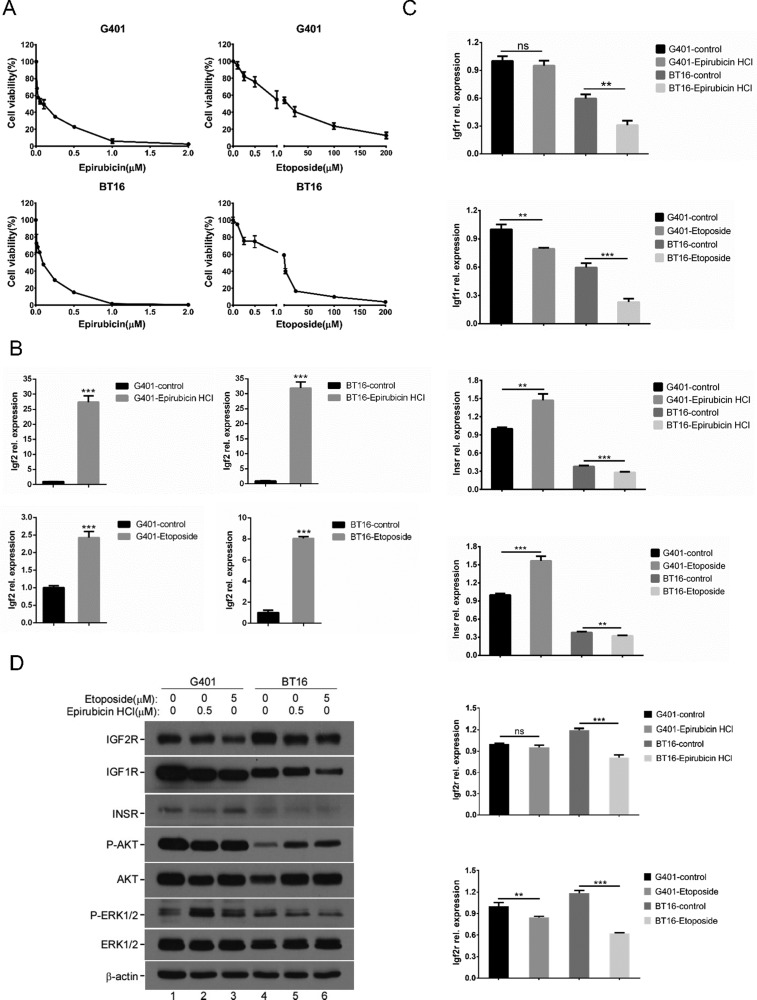
Chemotherapeutic agents induced IGF2 axis dysregulation (**A**) The effects of chemotherapeutic agents epirubicin HCl and etoposide on viabilities of G401, BT16. Cells were incubated with epirubicin HCl (0, 0.01, 0.025, 0.05, 0.1, 0.25, 0.5, 1, 2 μM) or etoposide (0, 0.1, 0.25, 0.5, 1, 5, 25, 100, 200 μM) for 72 hours, their viabilities were determined using MTS assays. Data represent the mean ± SD of three independent experiments. (**B**) The mRNA expression level of IGF2 following epirubicin HCl (0.5 μM) or etoposide (5 μM) exposure for 72 hours in G401 and BT16 were investigated by RT-qPCR. A representative example from 3 independent experiments is shown. Data represent the mean ± SD. *p < 0.05, **p < 0.01, ***p < 0.001. (**C**) RT-qPCR analysis of IGF1R, INSR and IGF2R mRNA levels. G401 and BT16 cell lines were treated with epirubicin HCl (0.5 μM) or etoposide (5 μM) for 72 hours. A representative example from 3 independent experiments is shown. Data represent the mean ± SD. *p < 0.05, **p < 0.01, ***p < 0.001. (**D**) G401 (lane 1-3) and BT16 (lane 4-6) were treated with epirubicin HCl (0.5 μM) or etoposide (5 μM) for 72 hours. The expression levels of IGF1R, INSR, IGF2R, and the activities of AKT and ERK1/2 were detected by western blotting analysis. β-actin served as a loading control. A representative example from 3 independent experiments is shown.

Chemotherapeutic treatment induces oxidative stress and contributes to drug resistance. There remains a critical need for effective strategies to overcome clinical chemotherapeutic agent resistance [16]. Evidence implies IGF2 is involved in the regulation of chemotherapy resistance and tumorigenicity. According to previous reports, IGF2 expression increased during prostate cancer progression in clinical databases [33]. In addition, following Taxol treatment, IGF2 mRNA levels progressively increased in ovarian cancer [16]. Considering our serum-free growth results, we next assessed whether chemotherapeutic agent exposure also affects the IGF2 axis in MRT. We randomly chose epirubicin HCl and etoposide which are chemotherapeutics commonly used to treat MRT. We confirmed the induction of cell death by epirubicin and etoposide in G401 and BT16 by MTS assay. Epirubicin and etoposide suppressed cell proliferation in a dose-dependent manner (Figure 2A). RT-qPCR results showed IGF2 mRNA levels progressively increased in both G401 and BT16 cells following chemotherapeutic treatment for 72 hours (Figure 2B). This was particularly significant in epirubicin, which increased IGF2 mRNA expression levels nearly 30-fold compared with untreated groups. Etoposide triggered a 2.5-fold (G401) and 8-fold (BT16) increase in IGF2 mRNA levels. In human, IGF2 promoters P2, P3, and P4 are active in many fetal tissues and then are down-regulated post-natally. P1 drives a low level of IGF2 expression in the adult liver [8]. As shown in Supplementary Figure 1, epirubicin upregulated the IGF2 mRNA level mainly through re-activated the P3 and P4 promoters. Unlike IGF2, mild changes were seen in IGF2 receptors, IGF1R, INSR and IGF2R (Figure 2C). IGF1R mRNA showed a slight reduction in both cell lines upon epirubicin and etoposide stimulation. INSR mRNA levels slightly increased relative to untreated control group in both cell lines when treated by epirubicin. But mildly reduced upon etoposide induction. The mRNA expression of IGF2R showed little decrease or no changed upon chemotherapy agent treatment. The low IGF2R expression was closely associated with the chemotherapy response in NSCLC patients. Patients with low IGF2R expressions had a poorer prognosis than those with high IGF2R expressions [34]. We also detected IGF1R, INSR and IGF2R protein levels by western blot. epirubicin and etoposide stimulation did not trigger obviously changes or only caused slightly reductions of IGF1R, INSR or IGF2R in G401 and BT16 (Figure 2D). The epirubicin and etoposide activated IGF2 axis could further activate downstream pathways. The phospho-ERK1/2 level increased in G401 cells, but the phospho-AKT level was not changed. On the contrary, the phospho-AKT level was increased obviously in BT16, but the phospho-ERK1/2 level was reduced slightly. Notably, the protein level of AKT was also increased in BT16 cells treated by epirubicin and etoposide (Figure 2D). The upregulation of AKT in BT16 was also confirmed by RT-qPCR (Supplementary Figure 2). These results support the idea that IGF2 plays an important role in the resistance to apoptotic stimulation from microenvironment *in vitro*.

### Autocrine IGF2 supported persistent growth of MRT cells by activating downstream multiple kinases signaling

**Figure 3:**
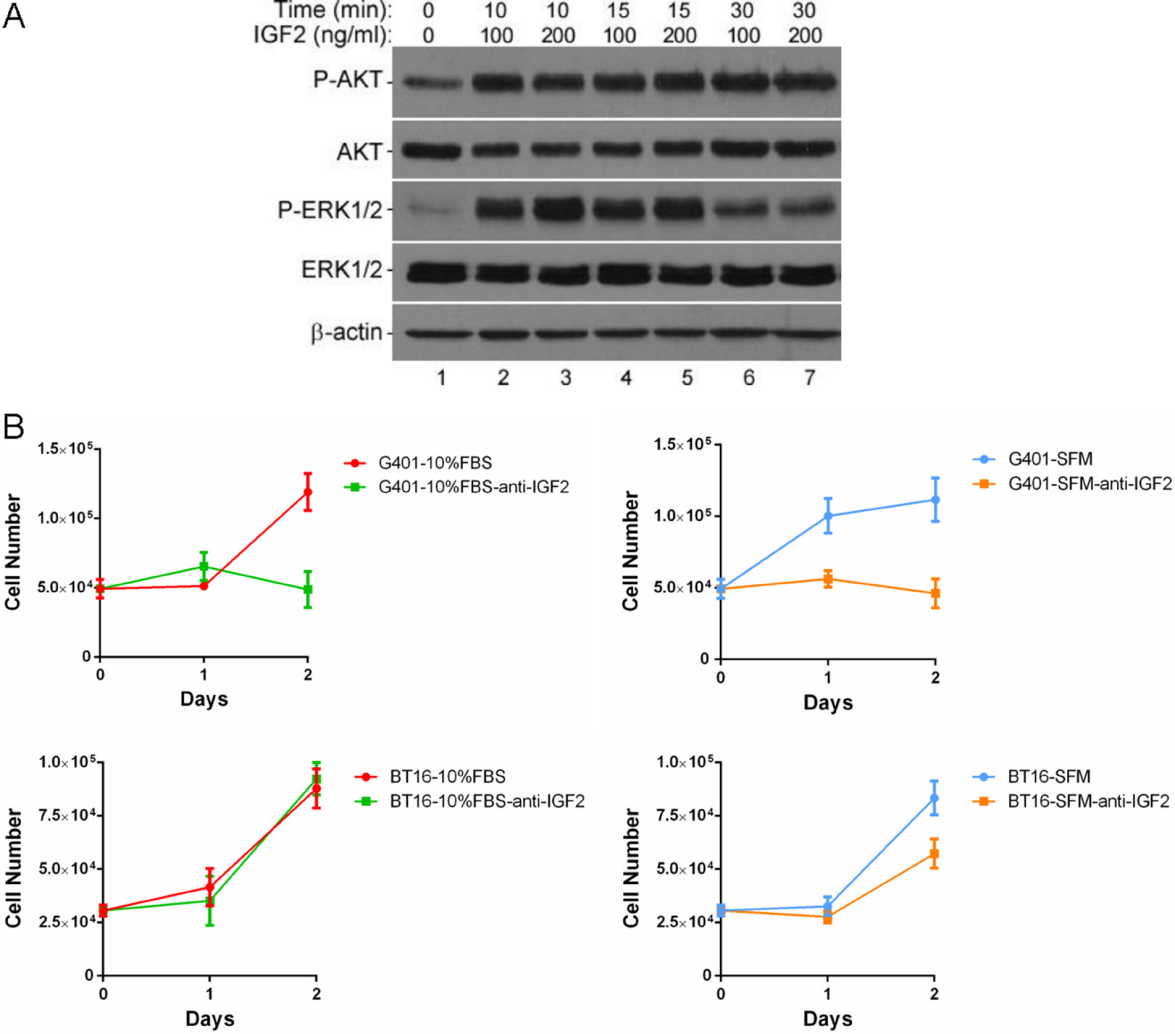
IGF2 activated a multiple kinases program, inhibition of IGF2 reduced cell viabilities in MRT cell lines (**A**) Effects of rhIGF2 on downstream signaling in G401 cells. Cells were starved for 12 hours, medium was changed again 2 hours before stimulation. Cells were treated with vehicle control, 100 or 200ng/ml rhIGF-2 under serum-free conditions for 0, 10, 15, or 30 minutes. The phosphorylation of AKT and ERK1/2 was detected by western blot analysis. β-actin served as a loading control. A representative example from 3 independent experiments is shown. (**B**) The anti-IGF-2 neutralizing antibody abolished the persistent growth of G401 and BT16. G401 and BT16 cells were cultured with α/Ham medium containing 10% FBS or serum-free α/Ham medium in the presence or absence of anti-IGF2 immunoglobulin (10 μg/ml) in 24-well dishes for 48 hours. Then the total cell number was counted on each day in a haemocytometer. Each point was done in triplicates, and is represented as the mean ± SD.

We investigated possible downstream mechanisms underlying the IGF2-IGF1R/INSR axis in MRT. The IGF1R and INSR activate phosphoinositide 3 kinase (PI3K) and mitogen-activated protein kinase (MAPK) signaling. These two pathways predominantly mediate cell survival and stimulate cellular proliferation, respectively [35]. We observed increased phospho-AKT and phospho-ERK1/2 levels upon the stimulation of recombinant human IGF2 protein at 10 minutes in starved G401 (Figure 3A, panel 1 and 3, lane 2 and 3). IGF2 induced phosphorylation of AKT lasted more than 30 minutes (Figure 3A, panel 1, lanes 2–7). In comparison, phospho-ERK1/2 reduced after 30 minutes exogenous IGF2 signal exposure (Figure 3A, panel 3, lane 6 and 7). However, levels were higher than basal in serum-free medium (Figure 3A, panel 3, lane 1).

IGF2 is secreted by most tissues where it acts in an autocrine or paracrine manner to regulate cell growth, differentiation and metabolism. To determine the role IGF2 plays in MRT cell proliferation, we added the neutralizing antibody against IGF2 in conditional α/Ham FBS-containing or serum-free medium to block the functions of autocrine IGF2. The results show markedly decreased proliferation of G401 regardless of serum-containing or serum-free culture (Figure 3B, top panels). The cell numbers were not increased, if not reduced, 48 hours after adding neutralizing anti-IGF2 immunoglobulin in medium containing 10% FBS (10%FBS-anti-IGF2) or serum-free medium (SFM-anti-IGF2). Serum-independent proliferation of BT16 was also impaired by IGF2 blockade, although not as sharp as G401. However, the growth rate was not changed by the IGF2 neutralizing antibody in the serum-containing group (Figure 3B, bottom panels). These results support the idea that IGF2 plays a significant role in promoting proliferation and adaptation to poor conditions in MRT.

### Blocking IGF1R inhibited IGF2 signaling and MRT cell growth

**Figure 4:**
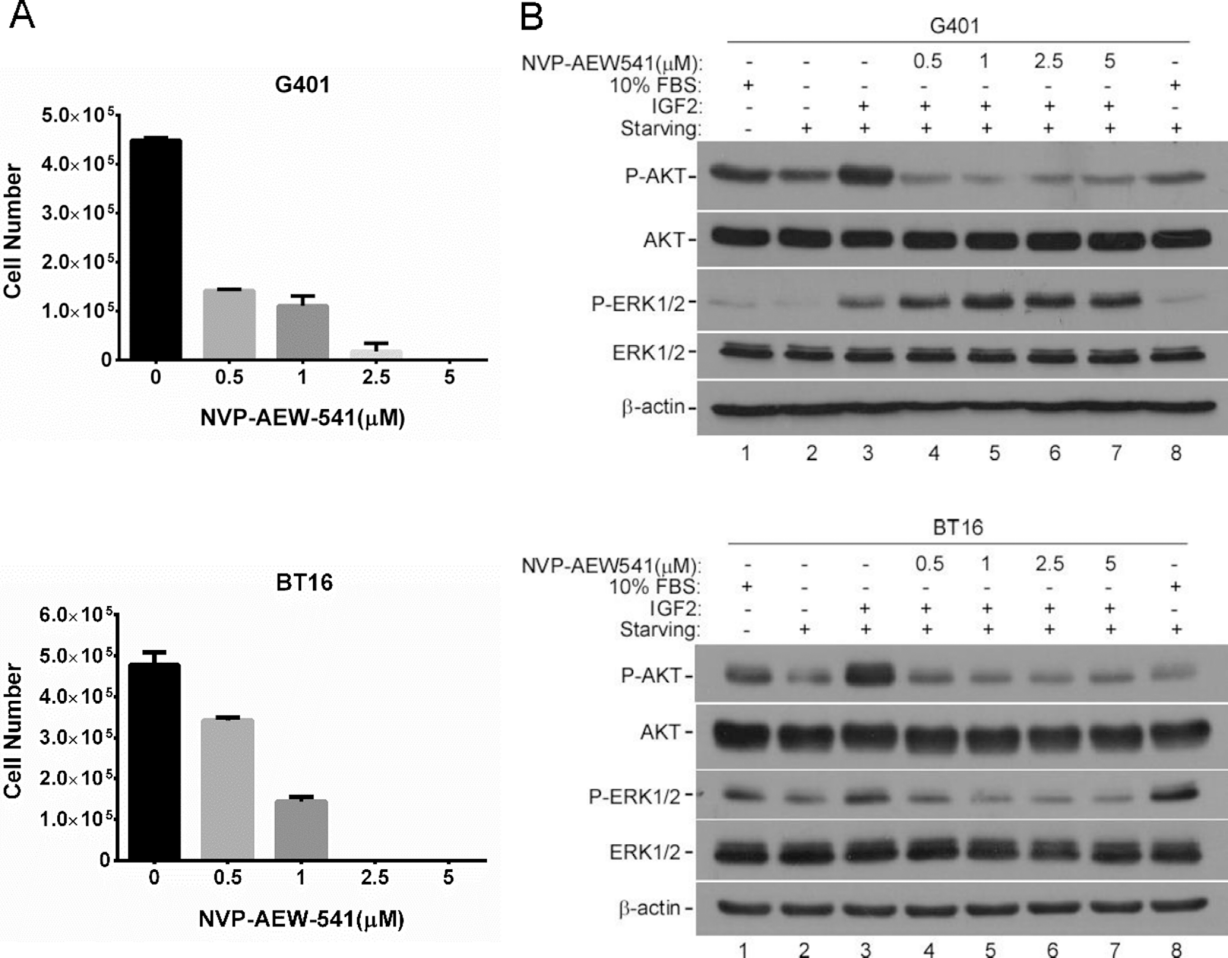
IGF2 activated downstream signaling mainly through IGF1R (**A**) IGF1R inhibitor NVP-AEW541 blocked the proliferations of G401 and BT16 cell lines. G401 and BT16 cells were cultured in α/Ham medium supplemented with 10% FBS containing DMSO or NVP-AEW541 at indicated concentrations (0.5, 1, 2.5, 5 μM). Triplicate dishes were counted in a haemocytometer at Day 3. Each point is represented as the mean ± SD. (**B**) Activities of downstream signaling in G401 and BT16 cells upon the treatment of NVP-AEW541 in absence or presence of IGF2. Cells were starved for 12 h, and medium was changed again 2 hours before stimulation. Vehicle control DMSO or IGF1R inhibitor NVP-AEW541 (0.5, 1, 2.5, 5 μM) was added 1 hour before rhIGF2 stimulation. Then G401 and BT16 cell lines were treated with 200 ng/ml rhIGF-2 or 10% FBS under serum-free conditions for 15mins. Activation of AKT and ERK1/2 were detected by western blot analysis. β-actin served as a loading control. A representative example from 3 independent experiments is shown.

In some childhood malignancies, IGF1R is activated by endocrine, autocrine, or paracrine mechanisms [36]. Biological functions of IGF2 are mainly exerted through IGF1R. Therefore, to investigate IGF1R as a mediator of IGF2 survival signaling, and as an potential therapeutic target in MRT, we determined whether blocking IGF1R inhibited tumor cell growth *in vitro* [30]. NVP-AEW541, a small molecule IGF1R tyrosine kinase inhibitor, was used to treat G401 and BT16 cell lines. This treatment caused a significant reduction of cell growth in G401 and BT16. In both cells, a dose dependent inhibition was clearly seen 3 days after treatment (Figure 4A). To determine whether IGF2-induced AKT and ERK1/2 phosphorylation are dependent on IGF1R activation, we evaluated the effect of a range of concentrations of NVP-AEW541 (0.5–5 μM) to disrupt IGF2 axis signaling in G401 and BT16 (Figure 4B) [16]. Cells were cultured in serum-free α/Ham medium for 12 hours, the medium was changed again 2 hours before rhIGF2 stimulation to avoid IGF2 autocrine accumulation. NVP-AEW541 was applied 1 hour before addition of rhIGF2. After stimulation with 200 ng/ml rhIGF2 or 10% FBS for 15 minutes, total cell lysate was collected, and western blot was performed to detect the IGF2-mediated downstream activation [37]. The phosphorylation levels of AKT and ERK1/2 showed a slight decrease after cells were starved for 14 hours compared with cells cultured in complete α/Ham medium (Figure 4B, lanes 1 and 2). The rhIGF2 activated AKT and ERK1/2 more effectively compared with 10% FBS in G401 and BT16 (Figure 4B, lanes 3 and 8). IGF1R inhibitor NVP-AEW541 effectively abrogated IGF2-induced AKT phosphorylation in both G401 and BT16, as well as ERK1/2 phosphorylation in BT16. We found no effects on phospho-ERK1/2 levels in G401 cells (Figure 4B, lane 4–7). Perhaps reduction of phospho-ERK1/2 needed a longer inhibitor exposure time, or IGF2 may activate the RAS/ERK pathway by INSR, which may compensate for the reduced IGF1R to transmit autocrine IGF2 signals, or other receptors as compensation when IGF1R was blocked in this cell line.

Interestingly, NVP-AEW541 reduced G401 cell viability cultured in α/Ham medium containing 10% FBS in a dose dependent manner (Supplementary Figure 3, red line). However, when the same assay was performed in serum-free α/Ham medium, we observed a slow decline of cell viability in concentrations ranging from 0.5 to 4 μM of NVP-AEW541 (Supplementary Figure 3, blue line). The percentage of cell viability in this concentration region ranged from 50.4% to 49.49% when cells were cultured in serum-free medium. This variation was more significant when cells were culture in complete medium, which was 56.65% to 28.94%. This phenomenon may be caused by increased IGF2, IGF1R and INSR in starved G401, but the accurate underlying mechanism requires further studies.

### AKT inhibitor impaired MRT viability *in vitro*

**Figure 5:**
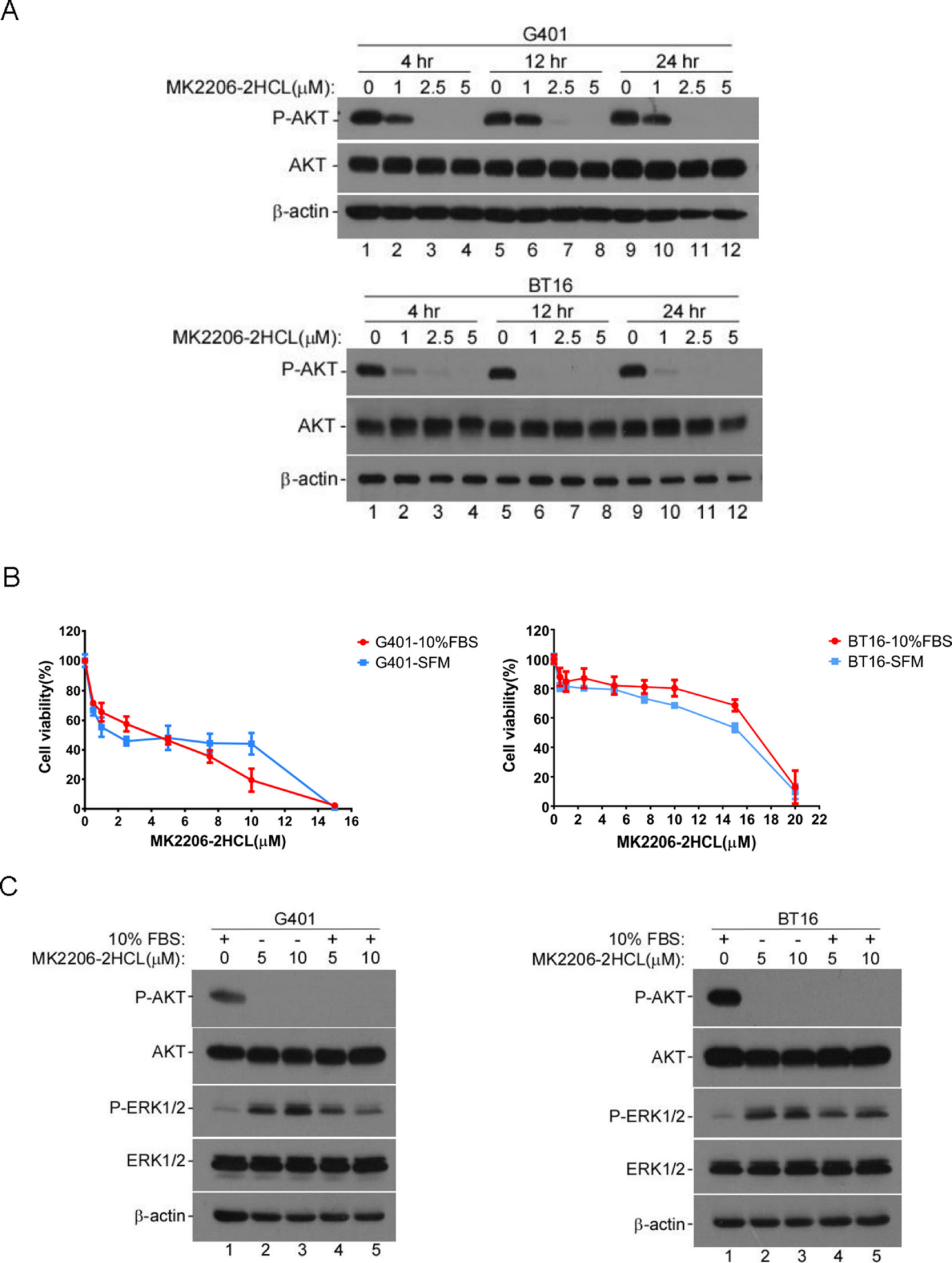
AKT inhibitor MK-2206 2HCL blocked the proliferation of MRT cells effectively (**A**) MK-2206 2HCl blocked AKT activation. G401 and BT16 cell lines were cultured in α/Ham medium containing 10% FBS. After attachment, dishes were washed and medium replaced with α/Ham medium containing 10% FBS supplied with DMSO or diverse concentration of MK-2206 2HCL as indicated (1, 2.5, 5 μM) for 4, 12, or 24 hours. The activity of AKT was detected by western blot. β-actin served as a loading control. A representative example from 3 independent experiments is shown. (**B**) The cytotoxicity of MK-2206 2HCL to G401 and BT16. Cells were seeded in 96-well plates, after attachment, medium was changed with α/Ham medium containing 10% FBS (-10% FBS) or serum-free medium (-SFM) supplied with serial dilutions of MK-2206 2HCL (0, 0.5, 1, 2.5, 5, 7.5, 10, 15 μM). After 72 hours, the cell viability was determined using MTS assay. Each point was done in triplicates, and is represented as the mean ± SD. (**C**) Western blot analyses of the effect of MK-2206 2HCl on AKT and ERK1/2 activities in G401 and BT16. G401 and BT16 were stimulated by MK2206 2HCl at indicated concentrations for 48 hours in the presence or absence of serum. β-actin served as a loading control. A representative example from 3 independent experiments is shown.

Previous studies have established the importance of PI3K/AKT signaling in transformation of many tumor types [38]. Reports show that AKT signaling pathway is a central axis in survival and proliferation of *smarcb1*-deficient cells [26]. Furthermore, according to our data, IGF1R inhibitor NVP-AEW541 blocks MRT cell growth accompanied by impaired AKT activity in G401 and BT16. To further validate the role of AKT signaling in survival and proliferation of MRT cells, we assayed the sensitivity of G401 and BT16 to a highly selective, pan-AKT inhibitor, MK-2206 2HCl. First, we confirmed this allosteric inhibitor effectively blocked AKT phosphorylation within 4 hours *in vitro* by western blot in G401 and BT16 (Figure 5A). As expected, inhibition of AKT activity reduced G401 and BT16 proliferation upon inhibitor treatment for 3 days (Figure 5B), indicating a dependence on PI3K/AKT signaling for MRT cell survival. Similar to NVP-AEW541, when G401 cells were cultured in serum-free medium, a stable cell surviving fraction appeared among the concentration from 2.5 to 10 μM. At this range, the percentage of cell viability varied from 50.09% to 46.21% (Figure 5B, blue line). This change was more dramatic in complete α/Ham medium group, showing a nearly linear decrease from 59.68% to 23.40% (Figure 5B, red line). Unlike G401, BT16 showed similar cell viability when cultured with or without serum. Furthermore, we evaluated the activity of RAS/ERK upon MK-2206 HCl treatment. surprisingly, the RAS/ERK pathway was activated by MK-2206 HCl, what is more, the expression levels of phospho-ERK1/2 were even higher in the serum-starved cells than non-starved cells, special in G401. Combined with the results shown in Figure 4B, targeting multiple pathways by means of combination treatment may be a more beneficial approach to treat MRT.

## DISCUSSION

**Figure 6:**
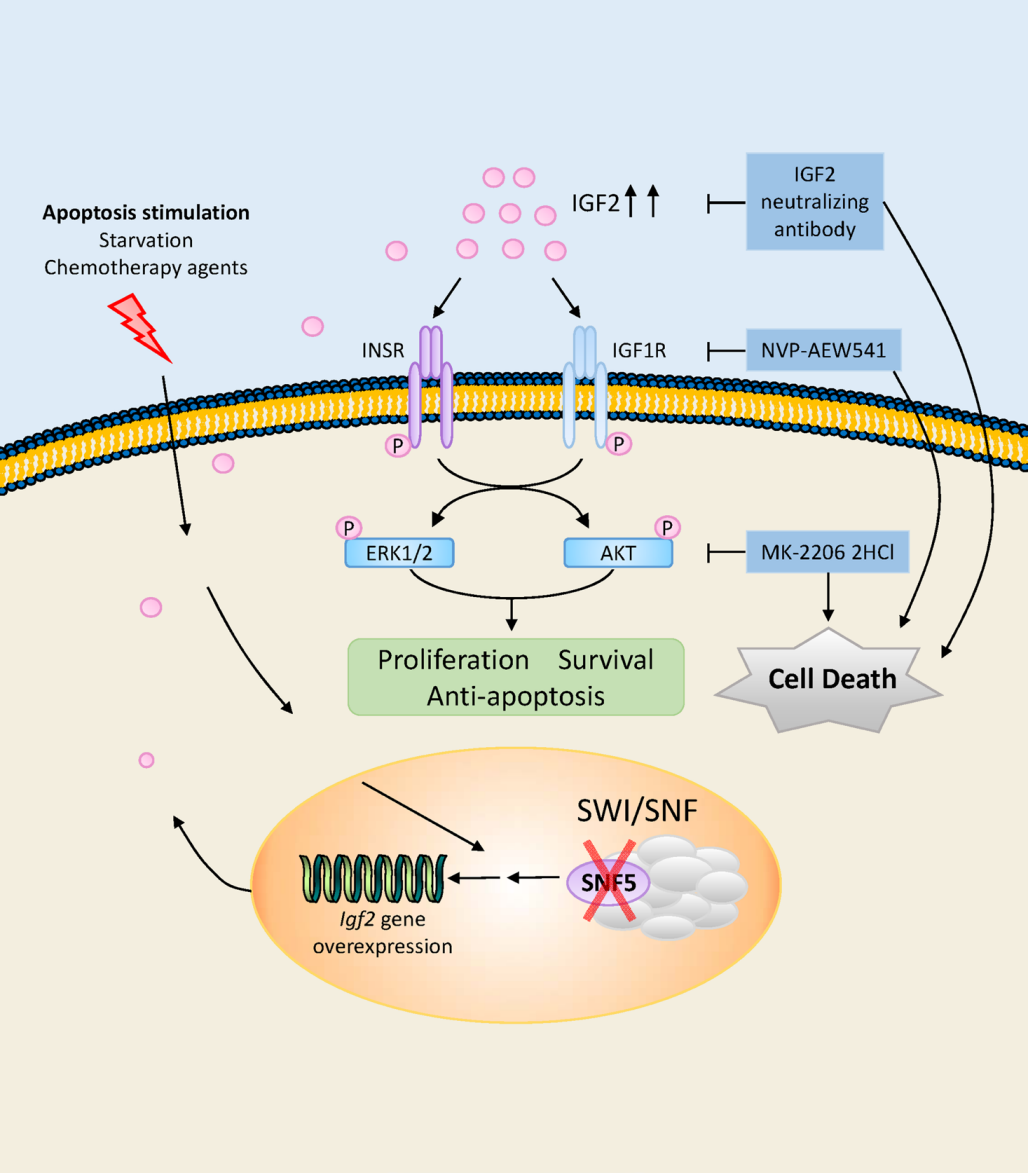
Schematic illustration of IGF2 axis, downstream kinase pathways, potential therapeutic targets in MRT cells In the SNF5/INI1/SMARCB1/BAF47 deficient MRT cells, IGF2 was upregulated when cells suffer from microenvironmental stress including serum deprivation and chemotherapeutic agent exposure. The autocrine IGF2 in turn activated intracellular PI3K/AKT and RAS/ERK pathways through IGF1R and INSR. These two pathways further contribute to survival, proliferation and anti-apoptosis of the tumor cells. Inhibiting this pathway, including IGF2 neutralizing antibody, IGF1R inhibitor NVP-AEW541 and AKT inhibitor MK-2206 2HCl, effectively abolished the ability of the tumor cells to grow *in vitro*.

MRTs are one of the most aggressive pediatric tumors arising in very early childhood, with a median age of onset of 11 months. Most children die from the disease within 1 year of diagnosis despite the use of intensive therapies [39]. In this study, we provide the first evidence that environmental stresses, including serum deprivation and chemotherapeutic agent exposure induce IGF2 upregulation in malignant rhabdoid tumor cell lines. We evaluated the role of IGF2 signaling pathway in MRT cell proliferations, and also confirmed this hormone promotes cancer cell growth through stimulation of IGF1R and INSR, which is associated with activation of PI3K/AKT and RAS/ERK pathways. Our findings show treatment targeting IGF2 axis, including neutralization of IGF2, inhibition of IGF1R or AKT activity by small molecule inhibitors, significantly impaired MRT growth *in vitro* (Figure 6). Therefore, our results provide preclinical evidence that IGF2 axis supports the persistent growth of MRT under microenvironment stress, and make this axis an attractive research target in respect of novel therapeutic strategy of MRT in the future.

During cancer progression, as a consequence of rapid growth, poor vascularization and necrosis, cancer cells are frequently exposed to physiological stress conditions such as hypoxia and nutrient limitation [40, 41]. Tumor cells must adapt to such stresses in order to survive and proliferate. The tumor microenvironment causes upregulation of various genes to increase survival potential and tumor aggressiveness [28]. To mimic the poor nutrient supply microenvironmental conditions, we cultured MRT cell lines in serum-free medium, and confirmed the serum-independent growth abilities in two MRT cell lines, G401 and BT16. We found the IGF2 axis was upregulated in starved MRT cell lines. Reports showed chemotherapeutic drug treatment or radiation therapy also cause oxidative stress and contribute to drug resistance [40]. We randomly chose two widely used clinical anti-MRT chemotherapeutic agents, epirubicin and etoposide, to treat MRT cells. According to our results, although the basal level of IGF2 mRNA in G401 and BT16 were lower compared with HEK293T, upon treatment of chemotherapeutic agents, IGF2 mRNA levels were significantly upregulated in G401 and BT16 as a result of the re-activation of the fetal promoters P3 and P4. Increases were approximately 30-fold when treated with epirubicin, and 2.5-8 fold when treated with etoposide. Considering the effect of IGF2 in promoting cell growth, the results of serum withdraw and chemotherapeutic agent exposure assays indicated IGF2 plays an essential role in MRT cell survival in the hostile microenvironment. Chemotherapy induced IGF2 overexpression was also reported by other groups, including Domingo-Domenech et al. where IGF2 was abundantly secreted into the medium of chemotherapy-resistant cultures [33]. According to Grasso et al. [42] and Taylor et al. [43], IGF2 is upregulated during disease progression. Moreover, following Taxol treatment, IGF2 mRNA levels progressively increased over 24 hours in findings by Horwitz et al. [16]. IGF2 is overexpressed in a spectrum of human cancers, and is associated with a poor prognosis [30]. Elevated serum IGF2 is also associated with increased risk of developing various cancers including colorectal, breast, ovarian, testicular, liver, and resistance to anticancer regimens [44]. Several mechanisms of IGF2 overexpression have been described. For example, loss of imprinting (LOI) is a well-described mechanism of IGF2 overexpression. But biallelic expression would presumably only lead to a two-fold increase in mRNA. Moreover, LOI of IGF2 is not exclusive to cancer cells, it is also commonly observed in normal neonates and adult humans [7]. Thus, this may not fully explain why chemotherapy treatment increases IGF2 expression in our case. In addition, IGF2 expression may be deregulated by transcription factors, including GATA2 [33], E2f3 [8], and ZFP57 [45]. However, the mechanism of IGF2 upregulation in MRT cells caused by microenvironment stress is still unknown. Characterized by the loss of SNF5, a core subunit of the SWI/SNF complex, multiple oncogenic pathways are upregulated in MRT. But, further research is needed to determine whether the increased IGF2 expression upon apoptotic stimulation is associated with SNF5-deficientcy and the activity of residual BRG1-containing SWI/SNF complex.

As shown in this paper, IGF2 activates PI3K/AKT and RAS/ERK pathways in G401 and BT16. These two pathways are involved in cell proliferation, survival and anti-apoptosis. Our data reveal IGF2 neutralizing antibody effectively decreased the serum-independent proliferation of G401 and BT16. The most significant effect was seen in G401, which expressed higher IGF2 level and exhibited stronger proliferation capacity than BT16 in the serum-free culture. This result indicates the IGF2-dependent serum-free growth of MRT. To date, ligand-targeting approaches, such as the fully human IGF2 ligand-neutralizing antibody BI 836845, and IGF2-bispecific antibody m67 are in the preclinical stage of development [18, 46].

IGF2 may bind to both the IGF1R and INSR tyrosine kinases. Intratumoral overexpression of IGF2 may indicate the presence of an autocrine loop, implying addiction to IGF1R activation and a higher probability of response to agents that effectively target this receptor [44]. According to our data, starvation treatment did not reduce mRNA or protein levels of IGF1R and INSR in G401 and BT16, and the expression of IGF2R was also hardly changed, which functions to clear IGF2 from the cell surface to attenuate signaling. Epirubicin and etoposide treatment slightly decreased expression of IGF1R and INSR, in the meantime reduced the IGF2R level. The IGF1R-specific small-molecule tyrosine kinase inhibitor NVP-AEW541 effectively blocked G401 and BT16 proliferation in our cases, demonstrating small-molecule inhibitors and monoclonal antibodies that target the IGF1R may be an alternative therapeutic strategy for clinical treatment of MRT [16]. Indeed, anticancer reagents using neutralizing antibodies to target growth factors and receptors have emerged as a new class of effective therapeutics for human cancer [30]. It is worth noting that NVP-AEW541 only blocked AKT but not ERK1/2 phosphorylation in G401, indicating the activation of AKT is mainly dependent on IGF1R in both cell lines. Nevertheless, ERK1/2 may be activated by other pathways in G401. Because IGF2 targeting antibody inhibits ligand activation of both IGF1R and IR-A, IGF2 targeting treatment may offer better outcomes compared with IGF1R targeting or IGF1R/INSR targeting therapy. The latter may be invalidated by crosstalk between the INSR and IGF1R pathways, or cause hyperinsulinemia and hyperglycemia by suppressing insulin signal through INSR, respectively [20].

Based on the previously reports, the PI3K/AKT pathway is a crucial means of maintaining the survival and growth of MRT cells, and this pathway is dysregulated in MRT cells [29]. Accumulating evidence indicates that the PI3K/AKT pathway also plays an important role in tumor angiogenesis and decreases the response of tumors to radiation [27]. In light of our data, drugs inhibiting the PI3K pathway may be of great value in MRT therapy. The highly selective inhibitor of pan-AKT, MK-2206 2HCl, effectively blocked G401 and BT16 growth. Interestingly, MK-2206 stimulation increased the phospho-ERK1/2 level, special in the absence of serum. A stable cell surviving fraction appeared when G401 was treated by gradually increased doses of NVP-AEW541 and MK-2206 in serum-free medium. One possible explanation is that starved G401 produced more IGF2 and IGF2 receptors. IGF2 may have enhanced the cells survival capacity through IGF1R and/or INSR against these two inhibitors at certain concentration ranges. But the exact mechanism still needs to be determined by further research.

In summary, our results show that IGF2 axis is upregulated in the harsh microenvironment, and acts as a key driver in the proliferation of MRT. These data suggest IGF2 plays a crucial role in tumor aggressiveness and chemotherapy resistance. Our results add to the current knowledge of MRT, and identify IGF2 axis as a promising target for future researches related to therapeutic approaches for MRT.

## MATERIALS AND METHODS

### Cell lines and cell culture

The MRT G401 and BT16 cell lines were a gift from Charles W.M. Roberts (Department of Pediatric Oncology, Dana-Farber Cancer Institute, Boston, Massachusetts, USA). The human embryonic kidney HEK293T cell line was a gift from Yupeng Chen (Department of Biochemistry and Molecular Biology, Tianjin Medical University, Tianjin, China). G401 cells were maintained in McCoy's 5A medium (Neuronbc) containing 10% FBS (Celima) and antibiotics (10 units/ml penicillin and 10 μg/ml streptomycin) (HyClone, GE Healthcare Life Sciences), BT16 cells were maintained in RPMI-1640 medium (HyClone, HyClone, GE Healthcare Life Sciences) containing 10% FBS (Celima) and antibiotics (10 units/ml penicillin and 10 μg/ml streptomycin). HEK293T were maintained in DMEM medium (Hyclone, GE Healthcare Life Sciences) containing 10% FBS (GIBCO) antibiotics (10 units/ml penicillin and 10 μg/ml streptomycin). For serum-free growth assay, cells were cultured in 50/50 vol/vol αMEM/Ham's F-12 (α/Ham) medium (HyClone, GE Healthcare Life Sciences) supplemented with 10% FBS or 10 μg/ml holo- transferrin human (SIGMA), and antibiotics (10 units/ml penicillin and 10 μg/ml streptomycin).

### Growth assays

Serum-free growth assays were carried out as described by Schofield et al. [31]. Briefly, cells were split into 60 mm dishes at an appropriate density. For routine assays this was usually 1–4 × 10^5^ per dish, about 10% confluence. The plating medium was α/Ham medium containing 10% FBS. After attachment overnight, dishes were washed twice by PBS, and finally 4 ml serum-free α/Ham medium supplemented with 10 μg/ml holo- transferrin human was added. Cells in three dishes were harvested by trypsin, and counted in a haemocytometer as a starting number, noted as Day -1. Twenty four hours later, dishes were washed by PBS, and medium was changed again with α/Ham medium containing 10% FBS or 10 μg/ml transferrin, triplicate dishes was counted and noted as Day 0. Medium was changed daily, and cell numbers were counted on each successive day to Day 7 in triplicate (in the case of HEK293T, cell number were counted to Day 5 given more than 90% cell death on Day 5). For small molecule inhibitors study, 0.5–1.5 × 10^5^ cells were split into 24-well dishes. After attachment overnight, dishes were washed twice by PBS, and medium was changed again with McCoy's 5A medium for G401 or RPMI-1640 medium for BT16 supplemented with 10% FBS. Small molecule inhibitor NVP-AEW541 (Selleckchem) was added at 0, 0.5, 1, 2.5, 5 μM. After 72 hours, triplicate wells for each inhibitor concentration were trypsinized and counted in a haemocytometer. For IGF2 neutralizing antibody assay, 2–3 × 10^4^ cells were split into 24-well dishes. After attachment overnight, dishes were washed twice by PBS, and medium was changed with serum-free α/Ham medium containing 10 μg/ml transferrin or α/Ham medium supplemented with 10% FBS. 10 μl IGF2 neutralizing antibody (millipore, 05–166) was added daily. Triplicate wells were trypsinized and counted in a haemocytometer after 24 and 48 hours.

### *In vitro* apoptotic stimulation on IGF2 axis

To detect the effect of apoptotic-inducing factors on the activities of IGF2 axis, 0.5–1.5 × 10^6^ cells were seeded into 6-well dishes, or 1–2 × 10^6^ cells were seeded into 60 mm dishes. After attachment overnight, dishes were washed twice by PBS. For starvation treatment assay, the medium was changed with serum-free α/Ham medium containing 10 μg/ml transferrin or α/Ham medium supplemented with 10% FBS, then cells were harvested 1–4 days later. For chemotherapy drugs treatment assay, the medium was changed with McCoy's 5A for G401 or RPMI-1640 for BT16 medium supplemented with 10% FBS, in addition of DMSO (SIGMA), 0.5 μM Epirubicin HCl (Dalian Meilun Biological Technology), or 5 μM Etoposide (Dalian Meilun Biological Technology), then cells were harvested 3 days later.

### *In vitro* activities of IGF2, NVP-AEW541 and MK-2206 2HCl

0.5–1.5 × 10^6^ cells were cultured in 6-well dishes, after attachment, dishes were washed twice by PBS. For IGF2 stimulation assay, cells were applied with serum-free α/Ham medium containing 10 μg/ml holo-transferrin human for 12 hours. Medium was changed again 2 hours before IGF2 stimulation to avoid the accumulation of autocrine IGF2. 100 ng/ml or 200 ng/ml recombinant human IGF2 (rhIGF2) (Abcam, ab9575) was added to stimulate cells for 10, 15 or 30 minutes. Medium was discarded immediately, cells were washed twice by PBS and harvested by cell lysis buffer (50 mM Tris-HCl, pH 6.8, 8 M urea, 5% mercaptoethanol, 2% SDS, and protease inhibitor mixture). To detect the effect of NVP-AEW541 on IGF2 induced activation of downstream signaling pathway, cells were applied with serum-free α/Ham medium containing 10 μg/ml transferrin or α/Ham medium containing 10% FBS for 12 hours. Medium was changed again 2 hours before rhIGF2 stimulation to avoid the accumulation of autocrine IGF2. 1 hour before stimulation, DMSO or 0.5, 1, 2.5, 5 μM NVP-AEW541 was added. Cells were treated with 10% FBS or 200 ng/ml rhIGF2 for 15 minutes, the medium was discarded immediately, after cells were washed twice by PBS, they were harvested by cell lysis buffer. To detect the effect of MK-2206 2HCl on downstream signaling pathway, after attachment, cells were incubated with medium supplemented with 1, 2.5, 5 μM MK-2206 2HCL or DMSO for 4, 12, 24 or 48 hours. The medium was discarded, and cells were washed twice by PBS followed by harvested by cell lysis buffer.

### Quantitative real-time PCR

Total RNA was isolated using the TRIzol^®^ Reagent (ambion^®^ by life technologies) according to the manual. RNA quantity, purity, and integrity were evaluated using the NanoDrop 2000c spectrophotometer (Thermo Scientific) and agarose gel electrophoresis. Complementary DNA was synthesized from 1–2 μg of total RNA using the Transcriptor First Strand cDNA Synthesis Kit (Roche) in accordance with manufacturer's instructions. Primer sequences used for amplification experiments are shown below: IGF2 forward primer, 5′-CCGTGCTTCC GGACAACT-3′; IGF2 reverse primer, 5′-GGACTGCTTC CAGGTGTCATATT-3′; IGF1R forward primer, 5′-GGA GGAGAAGCCGATGTGTG-3′; IGF1R reverse primer, 5′-ACGTGCTTGGGCACATTTTCT-3′; INSR forward primer, 5′-TACCCCGGAGAGGTGTGT-3′; INSR reverse primer, 5′-TCGGGCCTCGTTTTGAACAT-3′; IGF2R forward primer, 5′-TGAGCATGGGAACGCCTAAG-3′; IGF2R reverse primer, 5′-AGGGTACAGCCATCCAT CCT-3′; GAPDH forward primer, 5′-TTGCCCTCAACGA CCACTTT-3′; GAPDH reverse primer, 5′-TGGTCCAGGG GTCTTACTCC-3′.

qPCR was performed on an ABI 7500 fast real-time PCR System (Applied Biosystems) with a FastStart Universal SYBR Green Master [Rox] (Roche). In each 20 μl reaction, 1 μl reverse transcription product was used with 250 nM of each primer and SYBR diluted in DNase/RNase-free water to 1 × final concentration. Melting curve analysis was done to confirm a single amplicon corresponding to the product size for each reaction. Fold-change in relative mRNA expression was calculated using the 2*^–ΔΔCt^* method.

### Western bolt analysis

Western bolt assay was performed as described previously [47]. Briefly, cells were lysed by sonication for 30 seconds on ice in cell lysis buffer (50 mM Tris-HCl, pH 6.8, 8 M urea, 5% mercaptoethanol, 2% SDS, and protease inhibitor mixture). After addition of 4 × SDS sample buffer and boiling at 100°C for 7 min, equal amounts of total protein were subjected to SDS-polyacrylamide gel electrophoresis and transferred to a polyvinylidene fluoride membrane (Immobilon^®^-P^SQ^, Millipore). The membranes were skim milk (BD) blocked at room temperature for 1 hour or at 4°C overnight then probed with appropriate antibodies as described in figure legends at room temperature for 3 hours or at 4°C overnight. Primary antibodies used were: SNF5/INI1/SMARCB1/BAF47 (BETHYL, A301-087A), AKT (cell signaling technology, #4691), phospho-AKT (cell signaling technology, #4060), ERK1/2 (cell signaling technology, #4695), phospho-ERK1/2 (cell signaling technology, #4377), IGF1R (cell signaling technology, #3027), INSR (cell signaling technology, #3025), IGF2R (ImmunoWay, YT5269) and β-actin (Santa Cruz, sc-47778). After secondary antibody (cell signaling technology, #7074, #7076) incubation for 1 hour at room temperature, the targeted proteins were detected using chemilunimescence (ECL, Vazyme) and exposure of blots to X-ray films.

### MTS assay

Cell viability was analyzed by CellTiter 96^®^ Aqueous Non-Radioactive Cell proliferation Assay (Promega Corporation, Madison, USA). Cells were seeded at a density of 1–2.5 × 10^4^ cells per well in 96-well dishes and cultured overnight or for 24 hours. Then the medium was removed and replaced with new medium containing vehicle control or drugs at a series of concentration. After 72 hours, medium was changed again with a mixed solution composed of 100 μl medium and 20 μl MTS/PMS solution per well. The plates were incubated for 1–2 hours at 37°C in incubator, and the amount of soluble formazan produced by cellular reduction of the MTS/PMS was measured on a microplate reader (BioTek, Synergy HT) at 490 nm.

### Statistical analysis

All data shown represent mean ± standard deviation (SD) from at least three independent experiments. The differences in mean values between two groups were analyzed by Student's tests (two-tailed) performed using GraphPad Prism software (Version 6). *P value* less than 0.05 were considered statistically significant. Three asterisks indicate a statistically significant difference of *P* < 0.001, two asterisks indicate a statistically significant difference of *P* < 0.01, one asterisk indicates a statistically significant difference of *P* < 0.05.

## SUPPLEMENTARY MATERIALS FIGURES AND TABLES


