# Supplementary material for: Insulin-like growth factor 2 axis supports the serum-independent growth of malignant rhabdoid tumor and is activated by microenvironment stress
Source: Oncotarget. 2017 May 4;8(29):47269–83. doi: 10.18632/oncotarget.17617 (PMC5564563; doi:10.18632/oncotarget.17617)
Supplement: Supplementary file 1 [file oncotarget-08-47269-s001.pdf]

Insulin-like growth factor 2 axis supports the serum-independent growth of malignant rhabdoid tumor and is activated by microenvironment stress

SUPPLEMENTARY MATERIALS

MATERIALS AND METHODS

RT-qRCR was performed as described in the body of the paper. For the examination of IGF2 promoter usage, PCR products were analyzed by 2% agarose gel electrophoresis. Primer sequences used for RT-qPCR in supplemental experiments are shown below:

IGF2 P1 forward primer, 5′-CAGTCCTGAG GTGAGCTGCTGTGGC-3′ IGF2 P2 forward primer, 5′-ACCGGGCATTGCCCCCAGTCTCC-3′ IGF2 P3 forward primer, 5′-CGTCGCACATTCGGCCCCCGCGA CT-3′ IGF2 P4 forward primer, 5′-TCCTCCTCCTCCTGC CCCAGCG-3′ IGF2 common reverse primer, 5′-CAGCA ATGCAGCACGAGGCGAAGGC-3′ AKT forward primer, 5′-TCCCCCAGTTCTCCTACTCG-3′ AKT reverse primer, 5′-TCCCTCCAAGCTATCGTCCA-3′

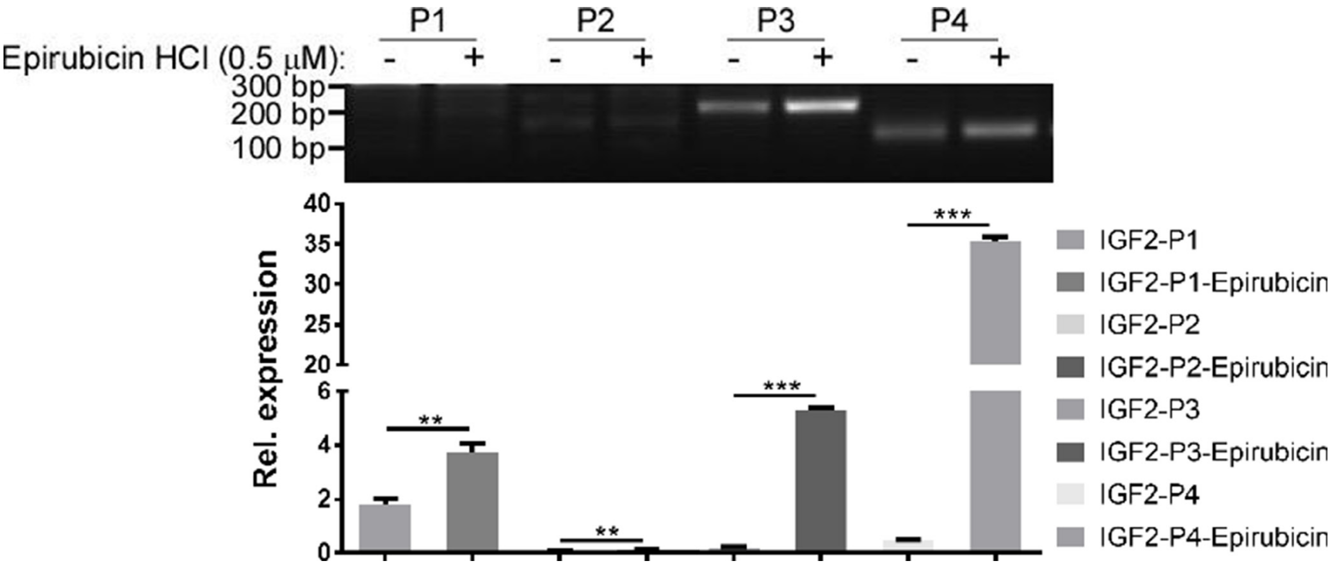

Supplementary Figure 1: The fetal promoters P3 and P4 were activated by chemotherapeutic agent stimulation. G401 cell was treated by 0.5 μM Epirubicin HCl for 72 hours, the usage of IGF2 promoters was detected by RT-qPCR, then the PCR products were analyzed by 2% agarose gel electrophoresis. A representative example from 3 independent experiments is shown. Data represent the mean ± SD. *$p < 0.05$, **$p < 0.01$, ***$p < 0.001$.

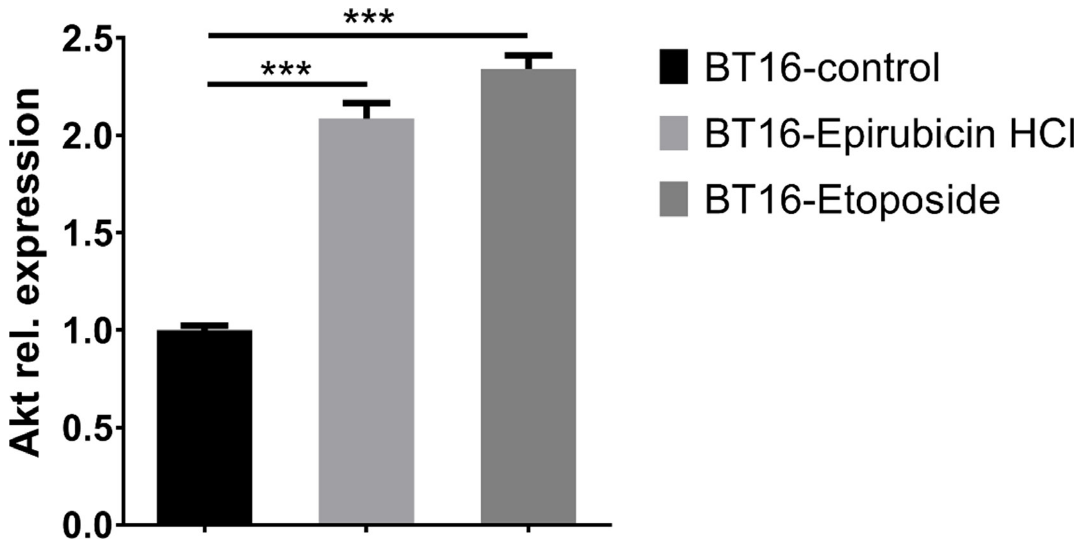

Supplementary Figure 2: AKT mRNA expression in BT16 samples with RT-qPCR. BT16 cells were treated by 0.5 μM Epirubicin HCl or 5 μM Etoposide for 72 hours, the expression level of AKT was detected by RT-qPCR. Data represent the mean ± SD. *$p < 0.05$, **$p < 0.01$, ***$p < 0.001$.

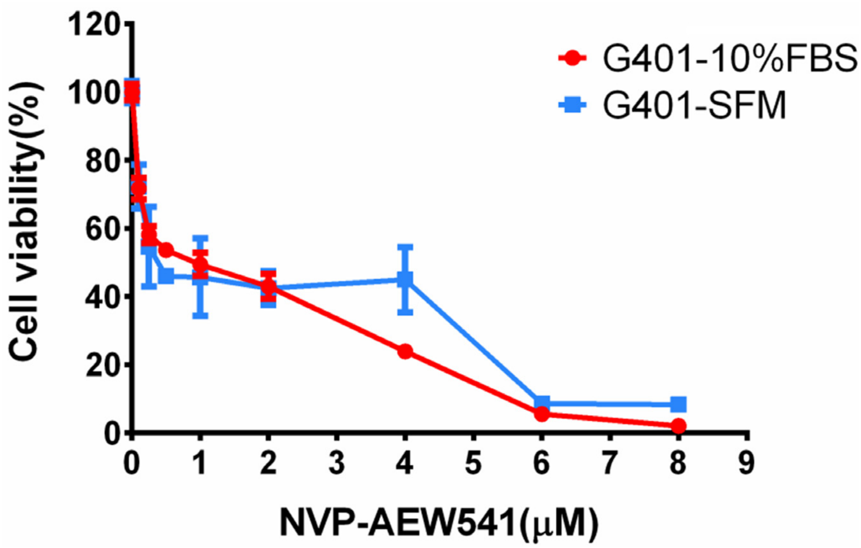

Supplementary Figure 3: The cytotoxicity of NVP-AEW541 to G401. Cells were seeded in 96-well plates, after attachment, medium was changed with α/Ham medium containing 10% FBS (-10% FBS) or serum-free medium (-SFM) containing serial dilutions of NVP-AEW541 (0, 0.1, 0.25, 0.5, 1, 2, 4, 6, 8 μM). After 72 hours, the cell viability was determined using MTS assay. Each point was done in triplicates, and is represented as the mean ± SD.
